# Activation of the canonical ER stress IRE1–XBP1 pathway by insulin regulates glucose and lipid metabolism

**DOI:** 10.1016/j.jbc.2022.102283

**Published:** 2022-07-19

**Authors:** Jinghua Peng, Caolitao Qin, Balamurugan Ramatchandirin, Alexia Pearah, Shaodong Guo, Mehboob Hussain, Liqing Yu, Fredric E. Wondisford, Ling He

**Affiliations:** 1 Department of Pediatrics, Johns Hopkins University School of Medicine, Baltimore, Maryland, USA; 2 Department of Nutrition, Texas A&M University, Texas, USA; 3 Division of Metabolism, Endocrinology and Diabetes, University of Michigan Medical School, Ann Arbor, Michigan, USA; 4 Division of Metabolism, Endocrinology and Nutrition, University of Maryland School of Medicine, Baltimore, Maryland, USA; 5 Department of Medicine, Rutgers-Robert Wood Johnson Medical School, New Brunswick, New Jersey, USA; 6 Departments of Pharmacology and Molecular Sciences, Johns Hopkins University School of Medicine, Baltimore, Maryland, USA

**Keywords:** XBP1, IRE1, insulin, AKT, liver glucose production, triglyceride, AAV, adeno-associated viruses, ACC2, acetyl CoA carboxylase 2, ATF, activating transcription factor, CREB, cAMP-response element-binding, ER, endoplasmic reticulum, IRE1, inositol-requiring enzyme, SCD1, stearoyl coenzyme desaturase 1, XBP1, X-box binding protein 1, XBP1u, XBP1 from an unspliced form, XBP1s, XBP1 from a spliced form

## Abstract

Knockout of the transcription factor X-box binding protein (XBP1) is known to decrease liver glucose production and lipogenesis. However, whether insulin can regulate gluconeogenesis and lipogenesis through XBP1 and how insulin activates the inositol-requiring enzyme-XBP1 ER stress pathway remains unexplored. Here, we report that in the fed state, insulin-activated kinase AKT directly phosphorylates inositol-requiring enzyme 1 at S724, which in turn mediates the splicing of XBP1u mRNA, thus favoring the generation of the spliced form, XBP1s, in the liver of mice. Subsequently, XBP1s stimulate the expression of lipogenic genes and upregulates liver lipogenesis as previously reported. Intriguingly, we find that fasting leads to an increase in XBP1u along with a drastic decrease in XBP1s in the liver of mice, and XBP1u, not XBP1s, significantly increases PKA-stimulated CRE reporter activity in cultured hepatocytes. Furthermore, we demonstrate that overexpression of XBP1u significantly increases cAMP-stimulated expression of rate-limiting gluconeogenic genes, *G6pc* and *Pck1*, and glucose production in primary hepatocytes. Reexpression of XBP1u in the liver of mice with XBP1 depletion significantly increases fasting blood glucose levels and gluconeogenic gene expression. These data support an important role of XBP1u in upregulating gluconeogenesis in the fasted state. Taken together, we reveal that insulin signaling *via* AKT controls the expression of XBP1 isoforms and that XBP1u and XBP1s function in different nutritional states to regulate liver gluconeogenesis and lipogenesis, respectively.

Activation of PI3K-AKT signaling by insulin plays a pivotal role in the regulation of glucose and lipid metabolism (1, 2). Activated AKT phosphorylates FOXO1 and TORC2, leading to nuclear extrusion and degradation (3, 4, 5, 6). We reported that phosphorylation of the CREB-binding protein at S436 by insulin disrupts the CREB–CBP–CTRC2 complex (7). Activation of PI3K-AKT signaling by insulin also upregulates *de novo* lipogenesis through mTORC and FOXO1 (8, 9, 10, 11). These insulin effects suppress liver gluconeogenesis and activate fatty acid synthesis. In the last decade, endoplasmic reticulum (ER) stress has emerged as a key player and nutrient sensor in the regulation of lipid and glucose metabolism because it can transduce some effects of metabolites into the activation of stress kinases (12, 13). ER stress leads to the cellular response termed the unfolded protein response through the activation of three canonical pathways: IRE1-XBP1, PERK-eIF2, and ATF6 (14, 15). X-box binding protein 1 (XBP1) is activated by the inositol-requiring enzyme (IRE1) in ER stress (16, 17, 18). Activated IRE1 splices out a 26-nucleotide intron from the XBP1 mRNA, leading to the shift in the codon reading frame. Translation of the new reading frame results in the conversion of XBP1 from an unspliced form (XBP1u) to a spliced form (XBP1s) that comprises the original N-terminal DNA-binding domain plus an additional domain in the C-terminus (17, 19).

Previous reports showed that mice with a liver-specific deficiency of XBP1 led to a reduction of gene expression related to lipid biosynthesis and triglyceride contents (20, 21). In contrast, liver overexpression of XBP1s upregulates lipogenesis by directly binding to the promoters of lipogenic genes, including stearoyl coenzyme desaturase 1 (SCD1) and acetyl CoA carboxylase 2 (ACC2) (20). Furthermore, XBP1s upregulates the transcription of fatty acid synthase genes by binding to the promoter of SREBP1 (22). These studies revealed an important role of XBP1s in the regulation of lipogenesis. Interestingly, conditional liver XBP1 KO mice exhibited significantly reduced fasting blood glucose levels and liver glucose production (21), indicating that XBP1 can modulate both glucose and lipid metabolism. Since liver glucose production and lipogenesis mainly occur during fasting and feeding, respectively, and knockout of XBP1 leads to the loss of both spliced XBP1s and unspliced XBP1u, it remains unclear whether XBP1u and XBP1s functions differently in the regulation of glucose and lipid metabolism. Furthermore, how the activation of IRE1-XBP1 signaling pathway occurs is also not well understood. Therefore, we determined whether nutritional status could affect the expression of distinct XBP1 isoforms and their roles in the regulation of glucose and lipid metabolism as well as the pathway, leading to the activation of IRE1-XBP1 signaling.

## Results

### The importance of XBP1 in the regulation of glucose and lipid metabolism in the liver

**Figure 1:**
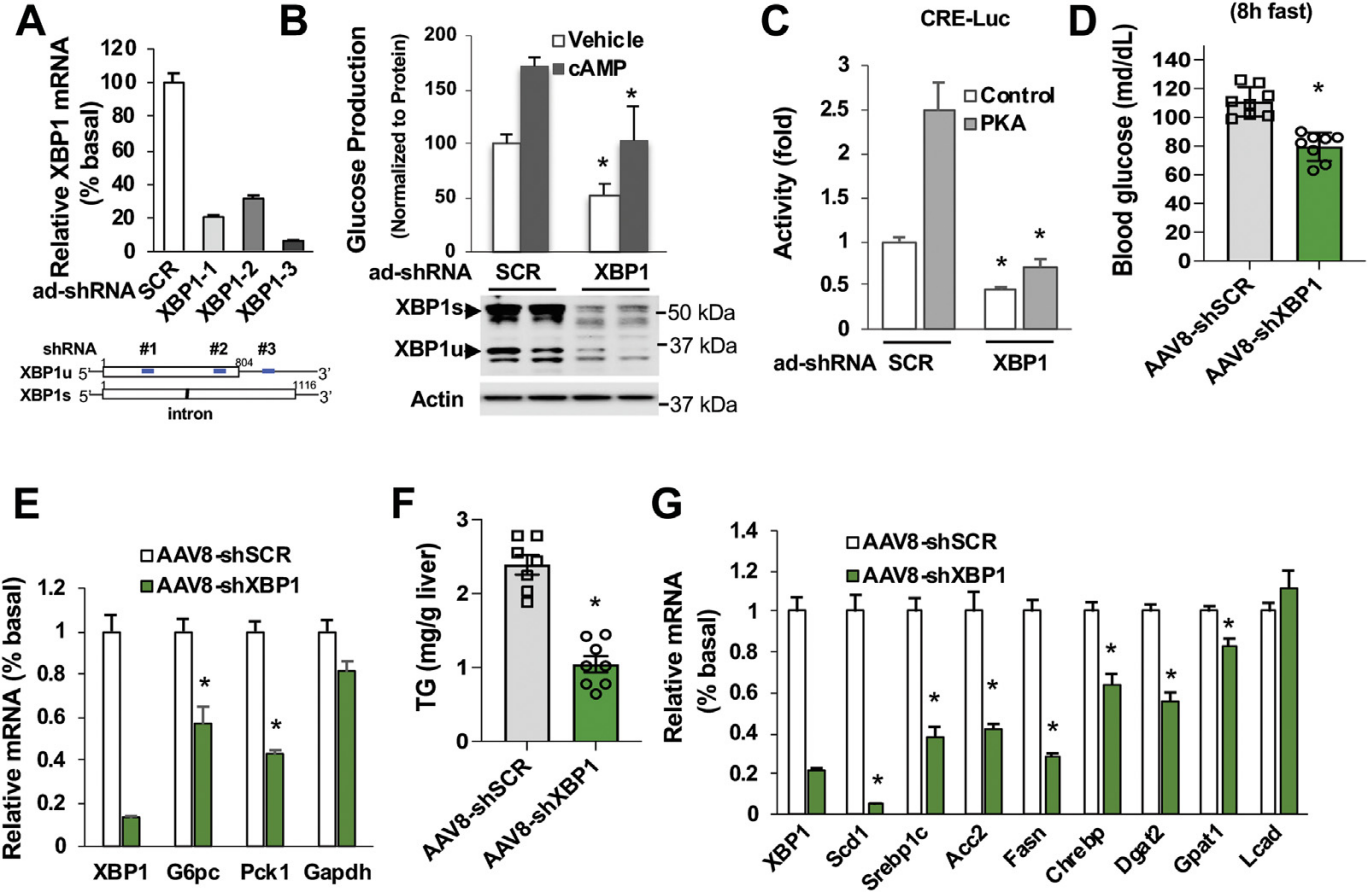
**XBP1 modulates glucose and lipid metabolism in the liver**. *A*, three sets of adenoviral shRNAs of XBP1 were added to Hepa1-6 cells for 48 h (n = 3). The *bottom panel* depicts the positions (*blue square*) of shRNAs’ targeting regions in XBP1. *B*, forty-eight hours after the addition of adenoviral shRNAs for scrambled control and XBP1 (ad-shXBP1-1), primary hepatocytes were subjected to serum starvation for 3 h, followed by glucose production medium supplemented with 0.2 mM cAMP for another 3 h (n = 3). *C*, adenoviral shRNAs for scrambled control and XBP1 (ad-shXBP1-1) were added 16 h after the seeding of Hepa1-6 cells. After 6-h incubation, 20 ng of reporter construct was transfected into Hepa1-6 cells together with 400 ng of control RSV-cat and PKA expression plasmids. Reporter activities were measured 48 h after the transfection (n = 3). *D* and *E*, three-month-old C57BL6 mice were injected with AAV8-scrambled shRNA or AAV8-XBP1shRNA (shXBP1-3) (3 × 10ˆ11GC/mouse). Sixteen days after the viral injection, blood glucose levels were examined after 8 h of fasting (*D*) (n = 8). Liver tissues were collected after 12 h of fast (*E*) (n = 5). *F* and *G*, three-month-old C57BL6 mice were injected with AAV8-scrambled shRNA or AAV8-XBP1shRNA through the jugular vein as in (*D*). Liver tissues were collected at 16 days after the viral injection, and triglyceride contents (*F*) (n = 7 ∼ 8) and the mRNA levels of genes related to lipid metabolism (*G*) were determined (n = 5). ∗*p* < 0.05, Student’s *t* test. AAV, adeno-associated viruses; SCD1, stearoyl coenzyme desaturase 1; XBP1, X-box binding protein 1.

Before studying the roles of different XBP1 isoforms in the regulation of liver metabolism, we first validated the importance of the XBP1 gene in regulating glucose and lipid metabolism by depletion of XBP1 mRNA in the liver. Three sets of adenoviral shRNAs were generated to deplete the XBP1 gene in Hepatoma 1-6 cells (Fig. 1*A*) and found that ad-shXBP1-1 and 1-3 had stronger efficacy; therefore, these sets of shRNAs were used in our subsequent studies. To assess the effect of XBP1 on glucose production in hepatocytes, we used adenoviral shRNA to deplete both XBP1 isoforms (XBP1u and XBP1s) in primary hepatocytes and found that depletion of XBP1 significantly reduced basal and cAMP-stimulated glucose production (Fig. 1*B*). Since XBP1 is a member of the cAMP-response element-binding (CREB)/activating transcription factor (ATF) ZIP family of transcription factors and can preferentially bind to the CRE site in the promoter of the target gene (23), we employed XBP1shRNA to deplete XBP1 in Hepa1-6 cells and conducted CRE-luciferase reporter assays to examine whether XBP1 could modulate gluconeogenic gene expression in hepatocytes. As shown in Fig. 1*C*, depletion of XBP1 significantly decreased basal and PKA-stimulated CRE-reporter activity. To further examine XBP1’s role in the regulation of glucose metabolism in the liver, the shRNA in the adenoviral vector (ad-shXBP1-3) was subcloned into the adeno-associated virus (AAV) expression vector, as we described previously (24, 25). The AAV8-XBP1shRNA was used to deplete liver XBP1 in three-month-old C57BL6 mice through jugular vein injection. XBP1 depletion led to a significant reduction in fasting blood glucose levels (Fig. 1*D*) and the mRNA levels of the rate-limiting gluconeogenic genes, *G6pc* and *Pck1* (Fig. 1*E*).

Similarly, mice with XBP1 depletion by AAV8-XBP1shRNA had significantly reduced triglyceride levels and the mRNA of genes related to lipogenesis in the liver (Fig. 1, *F* and *G*). These observations are in agreement with previous reports (20, 21) and collectively indicate that XBP1 can regulate both glucose and lipid metabolism in the liver.

### Refeeding activates the IRE1-XBP1s signaling

**Figure 2:**
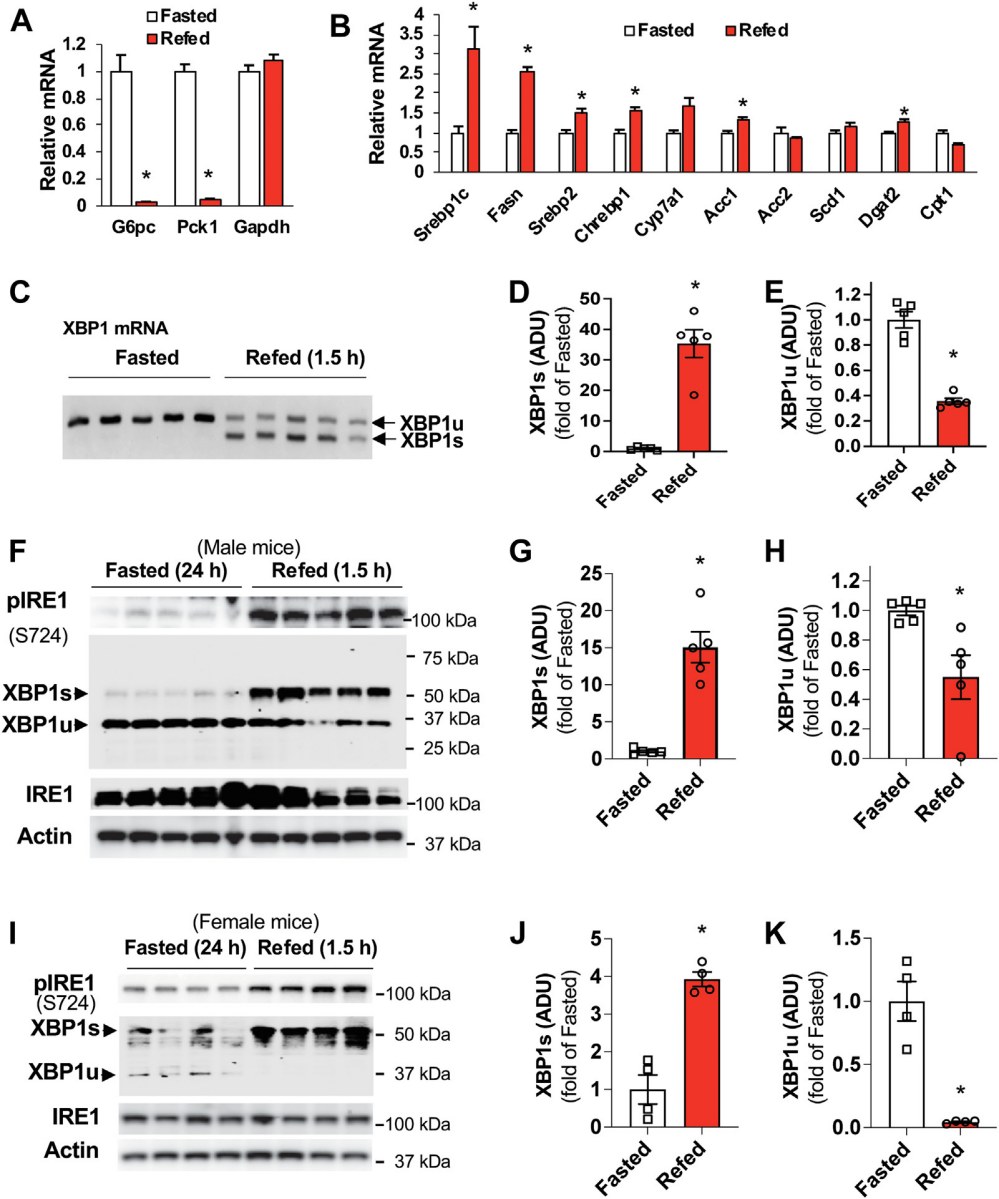
**Nutritional status affects the expression of XBP1 isoforms**. C57BL6 mice were subjected to a 24 h fast, then refed for 1.5 h. *A* and *B*, the mRNA of genes related to gluconeogenesis (*A*) and lipogenesis (*B*) in the livers of mice were collected in (*A*) (n = 5). *C–E*, the mRNA levels of unspliced and spliced XBP1 in the liver were determined by PCR (*C*). Densitometric analysis of the mRNA levels of XBP1s (*D*) and XBP1u (*E*) (n = 5). *F–H*, the protein levels of XBP1s and XBP1u in the liver of male mice (*F*) (each lane represents an individual mouse sample, n = 5). Densitometric analysis of the protein levels of XBP1s (*G*) and XBP1u (*H*). *I–K*, the protein levels of XBP1s and XBP1u in the liver of female mice (*I*) (each lane represents an individual mouse sample, n = 4). Densitometric analysis of the protein levels of XBP1s (*J*) and XBP1u (*K*). ∗*p* < 0.05, Student’s *t* test. *ADU*, Arbitrary Densitometric Units; XBP1, X-box binding protein 1; XBP1u, XBP1 from an unspliced form; XBP1s, XBP1 from a spliced form.

Since XBP1 depletion led to the decrease in blood glucose levels and the mRNA of gluconeogenic genes as well as the reductions of triglyceride contents and lipogenic gene expression in the liver (Fig. 1, *D*–*G*), we asked whether nutritional status could affect the expression of XBP1u and XBP1s. To test this hypothesis, 24-h fasted C57BL6 mice were refed for 1.5 h while the controls continued fasting. As expected, refeeding led to a suppression of gluconeogenic gene *G6pc* and *Pck1* expression, while lipogenic gene expression was significantly upregulated (Fig. 2, *A* and *B*). We determined the mRNA levels of XBP1u and XBP1s by targeting the region containing the 26-nucleotide intron because this intron would be spliced into the XBP1s (17, 19) and found that refeeding led to a drastic increase in the mRNA level of liver XBP1s that was suppressed in the fasted state. Furthermore, refeeding led to ∼65% reduction of liver XBP1u′s mRNA levels (Fig. 2, *C*–*E*). Refeeding increased the phosphorylation of IRE1 at S724 and XBP1s′ protein levels along with nearly 50% decrease in XBP1u′s protein levels in the liver of male mice (Fig. 2, *F*–*H*). Refeeding also significantly increased the phosphorylation of IRE1 at S724 and XBP1s′ protein levels and a more pronounced reduction of XBP1u′s protein levels in the liver of female mice (Fig. 2, *I*–*K*). Interestingly, the higher levels of XBP1u′s mRNA and protein are accompanied with higher expression of gluconeogenic genes in the fasted state, while the higher levels of XBP1s′ mRNA and protein are accompanied with higher expression of lipogenic genes in the refed state, suggesting that XBP1u and XBP1s may have a different function in the regulation of glucose and lipid metabolism in the liver.

### Activation of IRE1 by insulin modulates the expression of XBP1 isoforms in hepatocytes

**Figure 3:**
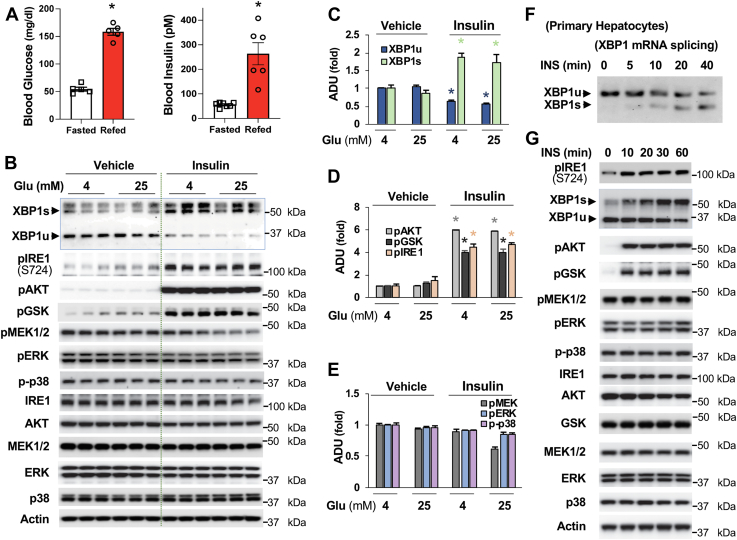
**Insulin activates IRE1-XBP1s signaling in cultured hepatocytes**. *A*, mice were subjected to a 24-h fast, then refed for 1.5 h, blood glucose levels (n = 5) and plasma insulin levels (n = 6 ∼ 7). *B–E*, Hepa1-6 cells were cultured in 4- or 25-mM glucose for 1 h, then treated with 10 nM insulin for 2 h. The protein levels of XBP1 and phosphorylation levels of mediators in the insulin signaling pathway and MEK-ERK-p38 signaling were determined (*B*). Densitometric analysis of the protein levels of XBP1s and XBP1u (*C*), phosphorylation levels of AKT, GSK, and IRE1 (*D*), and MEK, ERK, and p38 (*E*) (n = 3). *F*, primary hepatocytes were subjected to 3 h serum starvation, then treated with 10 nM of insulin for the indicated time. *G*, Hepa1-6 cells were subjected to 3-h serum starvation, then treated with 10 nM of insulin for the indicated time. ∗*p* < 0.05, Student’s *t* test. IRE1, inositol-requiring enzyme; XBP1, X-*box* binding protein 1; XBP1u, XBP1 from an unspliced form; XBP1s, XBP1 from a spliced form.

Having seen that refeeding activates the upstream kinase IRE1 and the expression of XBP1s in the liver and because refeeding results in the increase of both blood glucose and insulin levels (Fig. 3*A*), we reasoned that elevated blood glucose and/or insulin levels could activate the IRE1-XBP1 signaling and lead to an increase in XBP1s and a reduction of XBP1u. To test this notion, Hepa1-6 cells were treated with 4- or 25-mM glucose to mimic the low and high glucose concentrations, respectively, in the presence or absence of 10 nM of insulin for 2 h. We found that different glucose concentrations (4- or 25-mM glucose) did not affect the expression of XBP1u or XBP1s (Fig. 3, *B* and *C*). However, treatment with insulin led to increased IRE1 phosphorylation and XBP1s protein levels along with decreased XBP1u protein levels (Fig. 3, *B*–*D*). Moreover, in time course experiments, insulin promptly increased mRNA levels of XBP1s along with the reduction of XBP1u′s mRNA levels (Fig. 3*F*), and insulin also promptly augmented IRE1 phosphorylation and XBP1s protein levels in addition to a reduction of XBP1u′s protein levels (Fig. 3*G*). Since insulin can also activate the MEK–ERK–p38MAPK signaling pathway (1, 26), we found that the MEK–ERK–p38MAPK signaling pathway is activated in hepatocytes and treatment with either high concentration of glucose or insulin could not significantly change the phosphorylation levels of MEK, ERK, and p38MAPK (Fig. 3, *B*, *E*, and *G*).

### Insulin activates IRE1-XBP1 signaling in the liver of fasted mice

**Figure 4:**
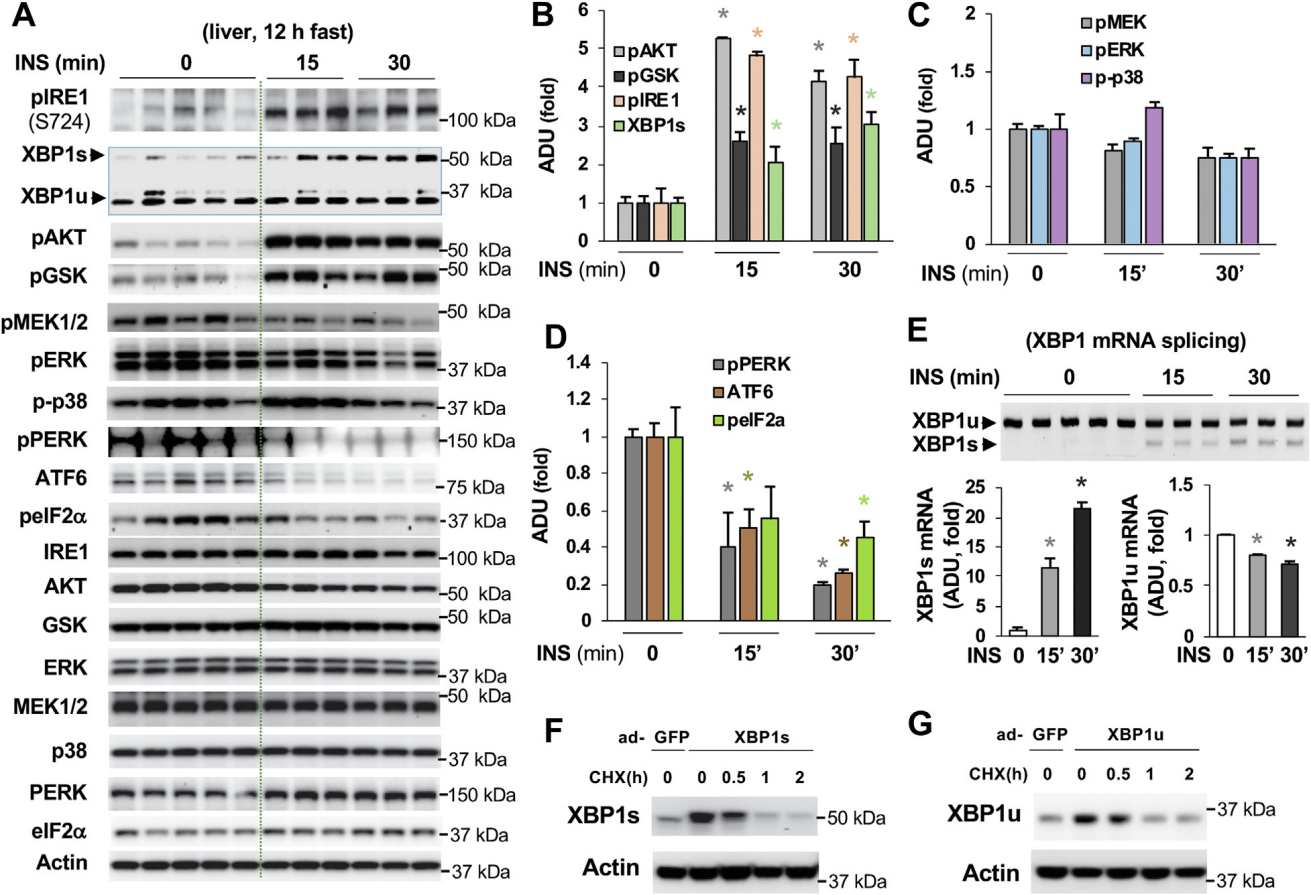
**Insulin activates IRE1-XBP1s signaling in the liver of fasted mice.***A–D*, after 12 h of fasting, mice were treated with vehicle or 1 unit/kg of insulin for 15 or 30 min, then liver tissues were collected. The protein levels of XBP1 and phosphorylation levels of mediators in the insulin signaling pathway, canonical ER stress pathway, and MEK-ERK-p38 signaling were determined in immunoblots (*A*). Densitometric analysis of the phosphorylation levels of AKT, GSK, IRE1, and protein levels of XBP1s (*B*), phosphorylation levels of MEK, ERK, and p38 (*C*), phosphorylation levels of pPERK and eIF2a and ATF6 protein levels (*D*) (n = 3 ∼ 5). *E*, the mRNA levels of XBP1s and XBP1u in the liver. Densitometric analysis of the mRNA levels of XBP1s and XBP1u (n = 3 ∼ 5). Each lane represents an individual mouse sample (*A*, *E*). *F* and *G*, forty-eight hours after the addition of adenoviral expression vectors of XBP1s or XBP1u, Hepa1-6 cells were treated with cycloheximide (CHX; 50 μg/ml) and harvested at the indicated time point. ∗*p* < 0.05, Student’s *t* test. ER, endoplasmic reticulum; IRE1, inositol-requiring enzyme; XBP1, X-box binding protein 1; XBP1u, XBP1 from an unspliced form; XBP1s, XBP1 from a spliced form; ER, endoplasmic reticulum.

To validate that insulin, not hyperglycemia, is able to activate the IRE1-XBP1 signaling, we treated 12-h fasted WT mice with 1 U/kg of insulin through intraperitoneal injection, and the liver tissues were collected 15 or 30 min after the insulin injection. We found that insulin treatment significantly increased IRE1 phosphorylation and XBP1s protein levels (Fig. 4, *A* and *B*) as well as the mRNA levels of spliced XBP1s along with the reduction of unspliced XBP1u′s mRNA levels (Fig. 4*E*). More pronounced changes were observed in the livers of mice 30 min after insulin treatment. These results indicate that insulin can indeed activate IRE1-XBP1 signaling. However, the protein levels of XBP1u was not affected by insulin (Fig. 4*A*), this may be due to the activation of counter-regulatory signaling, such as the elevation of blood glucagon (7) and a mild reduction of XBP1u′s mRNA as well as the half-life of the XBP1u protein being about 30 min (Fig. 4, *F* and *G*). Again, insulin could not augment the phosphorylation levels of MEK, ERK, and p38MAPK. On the contrary, insulin treatment led to decreases in the phosphorylation levels of MEK, ERK, and p38MAPK in the livers of mice after 30 min of insulin treatment (Fig. 4, *A* and *C*). In addition, insulin treatment negatively affected the PERK-eIF2 and ATF6 signaling because we observed decreased phosphorylation levels of PERK and eIF2α and ATF6 protein levels in the liver of mice treated with insulin (Fig 4, *A* and *D*).

### Activated AKT by insulin directly phosphorylates IRE1

**Figure 5:**
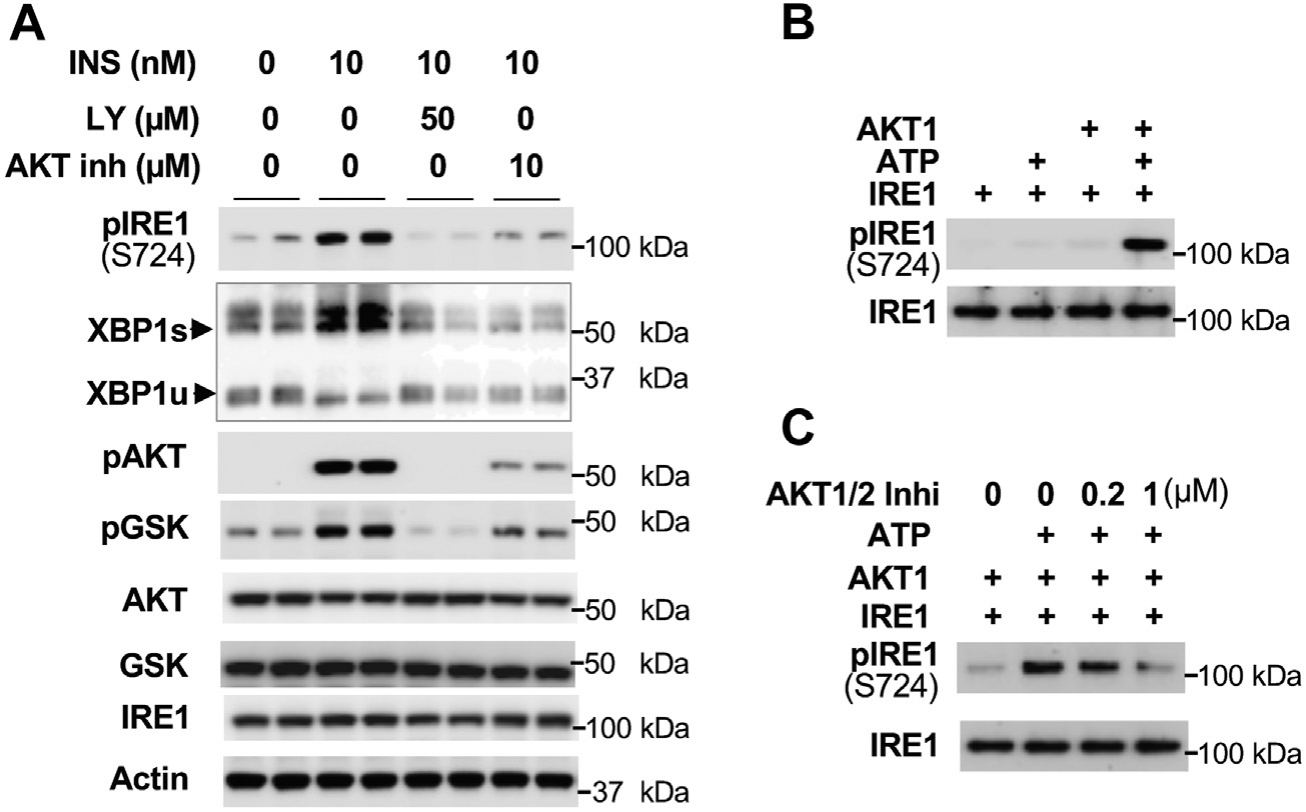
**AKT directly phosphorylates IRE1**. *A*, Hepa1-6 cells were cultured in Dulbecco’s modified Eagle’s medium without serum for 1 h, then 50 μM of LY294002 or 10 μM of AKT inhibitor II were added. After 1 h of incubation, 10 nM of insulin was added and cells were harvested 3 h later. Each lane represents an individual sample. *B*, 0.4 μg of IRE1 was incubated with 0.1 μg of AKT1 for 1 h at 37 °C in the presence or absence of ATP. The phosphorylation levels of IRE1 at S724 was examined using anti-IRE1S724 phosphorylation-specific antibody. *C*, AKT1/2 inhibitor was incubated with 0.4 μg of IRE1 for 10 min, then 0.1 μg of AKT1 was added and incubated for 1 h at 37 °C. The phosphorylation levels of IRE1 at S724 were examined as in (*B*). IRE1, inositol-requiring enzyme.

To determine the kinase that can phosphorylate IRE1, we used the PI3K inhibitor LY294002 and AKT inhibitor (Akt Inhibitor II) to block insulin signaling. These inhibitors negated insulin-stimulated IRE1 phosphorylation at S724 and generation of XBP1s protein as well as the reduction of XBP1u protein (Fig 5*A*), suggesting that activation of IRE1-XBP1 signaling by insulin is through PI3K-AKT signaling. Cloud-based NetPhos-3.1 identified IRE1 at S724 as a potential consensus AKT/PKB phosphorylation site (722-RHSF-725) (2). In an *in vitro* phosphorylation assay, using a phosphorylation-specific antibody against IRE1S724 (ab48187, abcam), we confirmed that AKT1 directly phosphorylated IRE1 at S724 in the presence of ATP (Fig. 5*B*), while IRE1 could not auto-phosphorylate at S724. Furthermore, pharmacologic inhibition of AKT kinase by the AKT1/2 inhibitor blunted AKT1-mediated IRE1 phosphorylation at S724 (Fig. 5*C*). These data suggest that insulin activates IRE1-XBP1 signaling through AKT phosphorylation of IRE1.

### Overexpression of XBP1s but not XBP1u augments lipogenic program in the liver

**Figure 6:**
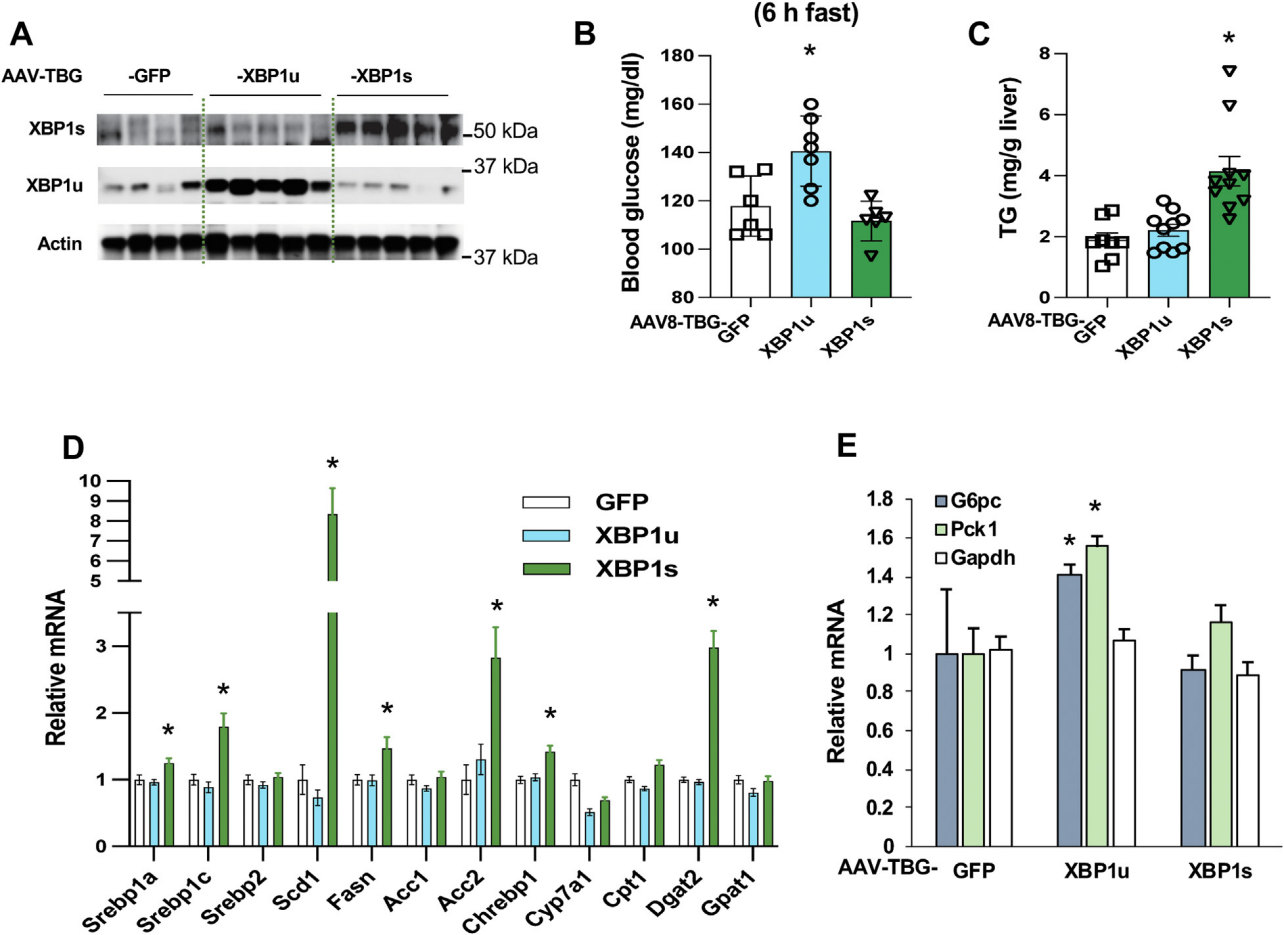
**XBP1s stimulates lipogenic gene expression**. C57BL6 mice were injected with AAV8-TBG-GFP, AAV8-XBP1u, and AAV8–XBP1s (1X10^12^ GC/mouse) through the jugular vein. Sixteen days after the viral injection, liver tissues of mice were collected during feeding state. *A*, protein levels of XBP1u and XBP1s in the liver (n = 4 ∼ 5). *B*, after 14 days of viral injection, blood glucose levels were determined (6 h fast) (n = 6 ∼ 7). *C–E*, the triglyceride contents (*C*) (n = 8 ∼ 10) and mRNA levels of genes related to lipid metabolism (*D*) (n = 5) and gluconeogenesis (*E*) were determined (n = 5). ∗*p* < 0.05, Student’s *t* test. AAV, adeno-associated virus; XBP1u, XBP1 from an unspliced form; XBP1s, XBP1 from a spliced form.

Depletion of XBP1(both XBP1u and XBP1s) led to significant reductions of triglyceride contents and the mRNA levels of lipogenic genes in the liver (Fig. 1, *F* and *G*). To test the effects of XBP1u and XBP1s on liver lipogenesis, we generated AAV8-TBG-expression vectors of XBP1u and XBP1s to express these proteins only in the liver. Using AAV-TBG-XBP1u and AAV-TBG-XBP1s expression vectors, ∼3-fold of either XBP1u or XBP1s was specifically expressed in the liver of C57BL6 mice (Fig. 6*A*), and liver tissues were collected in the fed state to test XBP1u and XBP1s′ effects on lipid metabolism. Overexpression of XBP1u had a minimal effect on triglyceride levels and did not augment the mRNA levels of lipogenic genes in the liver (Fig. 6, *A* and *C*). However, consistent with a previous report (20), overexpression of XBP1s led to a 2-fold increase in triglyceride contents and increased the mRNA levels of *SCD1* by over 8-fold and also significantly increased the mRNA levels of *SREBP1a* and *1c*, *Fasn, Acc2, Chrebp1*, and *Dgat2* (Fig. 6, *A* and *D*). These data indicate that XBP1s is the dominant XBP1 isoform in regulating lipid metabolism.

### XBP1u upregulates glucose production in hepatocytes

**Figure 7:**
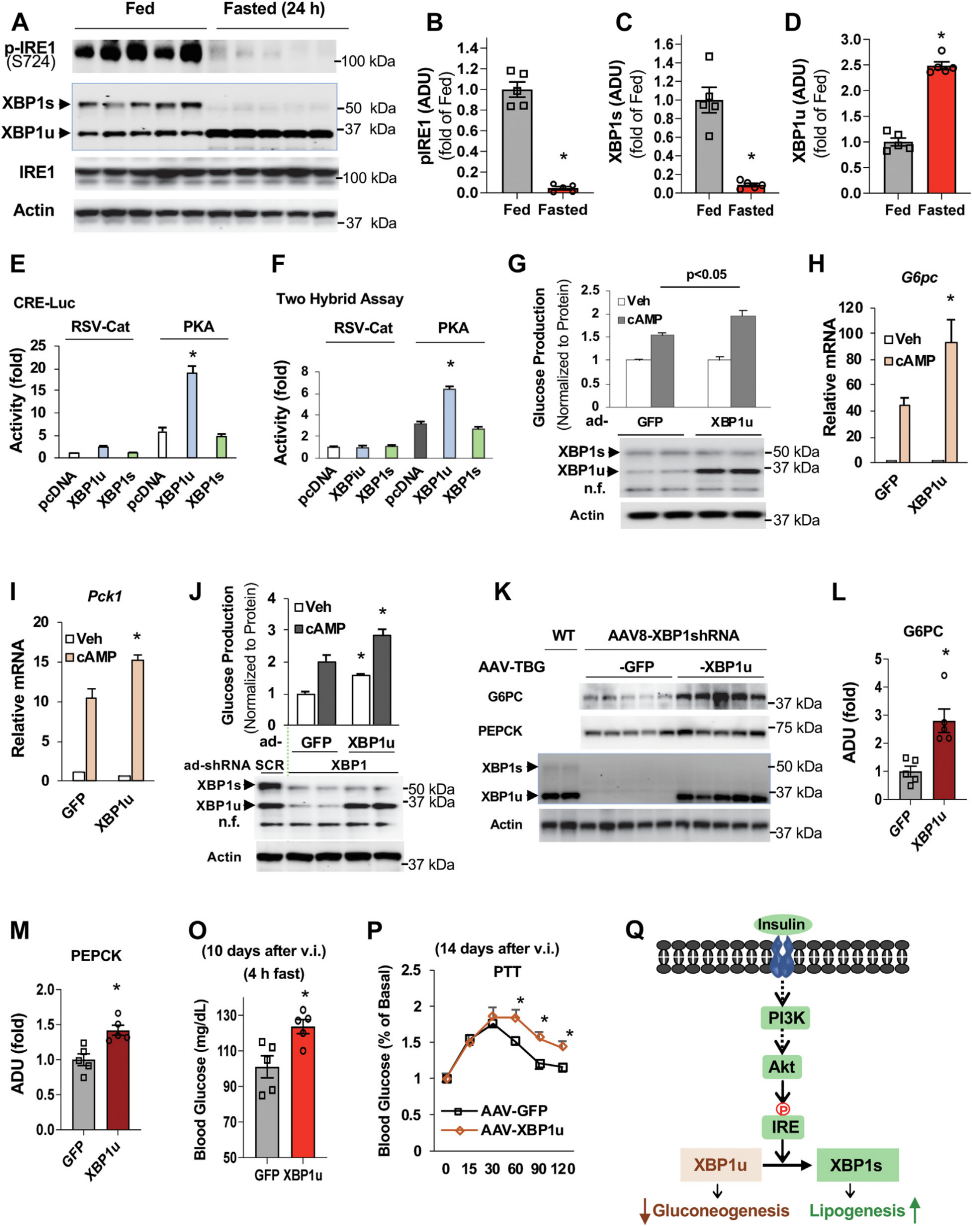
**XBP1u stimulates gluconeogenesis in cultured primary hepatocytes and liver.***A–D*, liver tissues of C57BL6 mice were collected at fed and fasted (24 h) states. Immunoblots (*A*), densitometric analysis of the phosphorylation levels of IRE1 (*B*), and protein levels of XBP1s (*C*) and XBP1u (*D*) (n = 5). *E*, XBP1u augmented PKA-stimulated reporter activity in Hepa1-6 cells cotransfected with 100 ng of XBP1u and XBP1s plasmids and 30 ng of CRE-Luciferase reporter plasmid (n = 3). *F*, Hepa1-6 cells were cotransfected with 150 ng of pFR-Luciferase reporter plasmid, 20 ng of pFA-CREB, 100 ng of XBP1u, XBP1s, and PKA plasmids (n = 3). *G–I*, twenty-four hours after the seeding of primary hepatocytes, adenoviral expression vectors of GFP and XBP1u were added. Forty-eight hours later, primary hepatocytes were subjected to 3 h serum starvation, followed by washing with PBS, and addition of glucose production medium and/or 0.2 mM cAMP for 3 h (*G*) (n = 3). One set of hepatocytes were harvested in TRIzol reagent for the determination of mRNA levels of *G6pc* (*H*) and *Pck1*(*I*) (n = 3). *J*, primary hepatocytes were treated with adenoviral shRNA of XBP1 together with adenoviral expression vectors of GFP or XBP1u. Cells were treated as in (*G*) (n = 3). *K–P*, three-month-old WT mice were injected (jugular vein) with AAV8-XBP1shRNA (shXBP1-3) (4 × 10ˆ11GC/mouse) and AAV8-TBG-GFP or AAV8-TBG-XBP1u (1.5 × 10^12^ GC/mouse) (n = 5). Immunoblots from collected liver tissues (*K*), and densitometric analysis of the protein levels of G6PC (*L*) and PEPCK (*M*). Ten days after the viral injection (v.i.), blood glucose levels were determined (4 h fast) (*O*) (n = 5). Fourteen days after the v.i., pyruvate challenge experiment was conducted (16 h fast, 1.5 g/kg) (*P*) (n = 5). Twenty-one days after the viral injection, liver samples were collected. Of note, two liver samples from 24-h fasted mice were loaded to serve as loading control in (*K*). *Q*, proposed model for insulin regulation of glucose and lipid metabolism through regulating IRE1-XBP1 signaling. n.f., nonspecific (*G* and *J*). ∗*p* < 0.05, Student’s *t* test. AAV, adeno-associated viruses; CREB, cAMP-response element-binding; IRE1, inositol-requiring enzyme; XBP1, X-*box* binding protein 1; XBP1u, XBP1 from an unspliced form; XBP1s, XBP1 from a spliced form.

Refeeding led to the reduction of liver XBP1u (Fig. 2, *F*, *H*, *I*, and *K*). In contrast, fasting suppressed the IRE1-XBP1 signaling, resulting in a 2.5-fold increase in XBP1u protein levels and a reduction of the expression of XBP1s (Fig. 7*A*). In addition, overexpression of XBP1u, not XBP1s, increased fasting blood glucose levels (Fig. 6*B*) and the mRNA levels of rate-limiting gluconeogenic genes *G6pc* and *Pck1* in the fed state (Fig. 6*E*). These aforementioned data suggest that XBP1u is the XBP1 isoform that may be able to upregulate gluconeogenesis in liver hepatocytes. To investigate whether XBP1u could affect the expression of the gluconeogenic gene, we first transfected XBP1u and XBP1s with the CRE-Luc reporter together with PKA expression plasmids and found that the cotransfection plasmid of XBP1u, not XBP1s, significantly increased reporter activity (Fig. 7*E*). In a Gal4-CREB two-hybrid assay, the cotransfection plasmid of XBP1u, not XBP1s, also significantly increased PKA-stimulated reporter activity (Fig. 7*F*).

Next, we used the adenoviral expression vector of XBP1u (Fig. 4*G*) and expressed 3-fold more of XBP1u than its endogenous protein levels in primary hepatocytes to test the effects on glucose production. Overexpression of XBP1u significantly increased cAMP-stimulated glucose production (Fig. 7*G*) and the mRNA levels of *G6pc* and *Pck1* genes (Fig. 7, *H* and *I*). To substantiate the role of XBP1u in upregulating glucose production in hepatocytes without a confounding effect of endogenous XBP1s, we used adenoviral shRNA-XBP1-3 (Fig. 1*A*) to target the sequence in the 3′ region of the XBP1 gene. This sequence does not exist in the coding region of XBP1u to deplete XBP1 in primary hepatocytes along with the expression of XBP1u to a similar level as its endogenous level in primary hepatocytes treated with adenoviral SCR (Fig. 7*J*). Compared with GFP-expressing control hepatocytes, XBP1u expression significantly increased basal and cAMP-stimulated glucose production in primary hepatocytes with XBP1 depletion (Fig. 7*J*).

AAV8-shRNA-XBP1-3 can deplete ∼90% of XBP1’s mRNA in the liver (Fig. 1*E*) *via* targeting the noncoding region of XBP1u, allowing us to efficiently deplete liver XBP1 while selectively expressing XBP1u using the AAV8-TBG-XBP1u expression vector. Using this approach, we achieved liver XBP1u levels comparable to those found in the liver of the 24-h fasted mice (Fig. 7*K*). Mice with selective XBP1u expression had significantly higher fasting blood levels than GFP-expressing control mice (Fig. 7*O*). Upon conducting the pyruvate challenge, mice with XBP1u expression produced more glucose than the control mice (Fig. 7*P*). Moreover, XBP1u-expressing mice had higher protein levels of G6PC and PEPCK in the liver (Fig. 7, *K*–*M*).

## Discussion

Insulin, a powerful anabolic hormone, regulates both glucose and lipid metabolism. In the feeding state, elevated blood insulin levels promptly stimulate *de novo* lipogenesis and triglyceride synthesis using carbons from amino acids, glycerol, or the spillover of glucose in the liver (27, 28). Since knockout of the liver XBP1 gene decreased lipogenesis (20, 21) and lipogenesis mainly occurs during feeding, we determined whether the nutritional status could affect IRE1-XBP1 signaling and found that refeeding activated IRE1 and led to the conversion of XBP1 expression from XBP1u to XBP1s in the liver. A previous study also showed that overexpression of XBP1s upregulates liver lipogenesis (20). These findings indicate that XBP1s is in the isoform that drives lipogenesis in the liver in the fed state.

To determine how IRE1-XBP1 signaling is activated in the fed state, we tested the effects of high glucose concentration or insulin on the phosphorylation of IRE1 as blood levels of glucose and insulin are significantly elevated during feeding. We found that it is insulin, not glucose, that stimulates the activation of IRE1-XBP1 signaling. This insulin effect on the activation of IRE1-XBP1 signaling has been proven in three ways. First, insulin treatment promptly stimulated the splicing of the XBP1 gene, leading to the generation of XBP1s mRNA levels along with the reduction of unspliced XBP1u mRNA levels in primary hepatocytes. Second, in hepa1-6 cells, insulin stimulated the generation of XBP1s protein while reducing XBP1u protein levels in a time-course experiment. Furthermore, insulin stimulated the generation of XBP1s′ mRNA and protein levels in the liver of fasted mice. These data indicate that insulin can upregulate lipogenesis through activation of IRE1-XBP1 signaling during feeding. More specifically, activated AKT by insulin directly phosphorylates IRE1 at S724 to mediate the splicing of the XBP1 gene and the generation of XBP1s. Previous studies showed that AKT plays a critical role in insulin-stimulated lipogenesis (8, 9, 10, 11). In line with these reports, our study reveals that overexpression of XBP1s leads to increases in the mRNA levels of *Srebp1c*, *Fasn*, and *Chrebp1* and had greater impacts on the mRNA levels of *Scd1, Acc2*, and *Dgat2.* However, activation of mTORC signaling by AKT more potently stimulates the expression of *Srebp1c* and *ATP*-citrate lyase genes (8, 9, 10, 11). These data suggest that activation of AKT-IRE1-XBP1 signaling works in concert with AKT-mTORC signaling to upregulate *de novo* lipogenesis and triglyceride synthesis.

Intriguingly, knockout of XBP1 also significantly decreased blood glucose levels in 14-h fasted mice (21). Consistent with this report, we found that depletion of liver XBP1 by AAV-shRNA significantly decreased fasting blood glucose levels and rate-limiting gluconeogenic gene expression. However, we found that in the fasted state, XBP1s’ protein levels are almost undetectable and XBP1u is the dominant XBP1 isoform as well as that fasting augmented XBP1u protein levels by 2.5-fold in the liver, suggesting that XBP1u is the isoform that upregulates gluconeogenesis. Indeed, in the liver and cultured primary hepatocytes with depletion of XBP1 (both XBP1u and XBP1s), selectively expressing XBP1u restored glucose production and rate-limiting gluconeogenic gene expression. Moreover, overexpression of XBP1u, not XBP1s, in the liver increased fasting blood glucose levels. Even though XBP1u and XBP1s have the same DNA-binding domain, XBP1u, not XBP1s, can stimulate the CRE-Luc reporter activity. Furthermore, expression of XBP1u, but not XBP1s, increases the Gal4-CREB reporter activity. We speculate that the shift in the codon reading frame and the addition of the extra domain in the C terminus of XBP1s may hinder the protein’s binding to the CRE site or form a complex with CREB (17, 19). In summary, the activation of IRE1-XBP1 signaling by insulin-activated AKT increases XBP1s to stimulate liver lipogenesis in the fed state, while in the fasted state, augmentation of XBP1u upregulates liver gluconeogenesis to maintain the euglycemia.

Of particular interest, in a human study (29), there is a 2-fold increase in XBP1s′ mRNA levels in the liver of obese patients with steatosis, and obese patients with fatty liver have elevated liver XBP1 protein levels. These human data suggest that elevated XBP1s may be a mediator driving the development of fatty liver in obese patients. However, more human studies are needed to define the roles of XBP1s and XBP1u in the development of hyperglycemia and steatosis in obese patients.

## Experimental procedures

### Plasmids, adenoviruses, and AAVs

The BLOCK-iT adenoviral RNAi expression system (Invitrogen) was used to construct adenoviral shRNAs for XBP1 and scrambled shRNA vectors, as we previously described (30). We found that the following three sets of sequences of XBP1shRNAs can efficiently deplete the XBP1 gene: 5′-GGAAGAAGAGAACCACAAACT-3′, 5′-GCCAAGCTGGAAGCCATTAAT-3′, 5′-GGGCATCTCAAACCTGCTTTC-3’. These vectors were also employed to generate AAV-vectors. Regions in the pENTR/U6 vector containing the U6 promoter, Pol III terminator, and XBP1shRNA oligo or scrambled shRNA oligo were amplified by PCR and cloned into the AAV-BASIC vector (Vector Biolabs); these vectors were used to make AAV8 shRNAs for XBP1 and scrambled shRNA. The coding regions of the mouse XBP1u and XBP1s genes were synthesized (GenScript), and these genes were subcloned into the pcDNA 3.1 Directional TOPO Expression Vector (Invitrogen). The pAAV-TBG-EGFP expression vector was purchased from Vector Core at the University of Pennsylvania. With permission, XBP1u and XBP1s genes were used to replace EGFP and generated pAAV-TBG-XBP1u and pAAV-TBG-XBP1s expression vectors.

### Animal experiments

All animal protocols were approved by the Institutional Animal Care and Use Committee of the Johns Hopkins University. All the male mice were used, unless otherwise specified. Three-month-old C57BL6 mice were injected with AAV8-scrambled shRNA or AAV8-XBP1shRNA (shXBP1-3) (3 × 10ˆ11GC/mouse) through the jugular vein. Sixteen days after the viral injection, blood glucose levels were examined after 8 h of fasting and liver tissues were collected after a 12-h fast. In another set of mice injected with the same amounts of AAV8-scrambled shRNA or AAV8-XBP1shRNA (shXBP1-3) (3 × 10ˆ11GC/mouse), liver tissues were collected during fed state after 16 days of viral injection. To test the effects of nutritional status on the splicing of the XBP1 gene, C57BL/6 mice at the age of 12 weeks were fasted for 24 h, then refed for 1.5 h. In an experiment to determine insulin-mediated activation of IRE1-XBP1 signaling, C57BL/6 mice were fasted for 12 h, followed by treatment with vehicle or 1 U/kg of insulin for 15 or 30 min. To test the effects of XBP1u and XBP1s on glucose and lipid metabolism, three-month-old C57BL6 mice were injected with AAV8-TBG-GFP, AAV8-TBG-XBP1u, and AAV8-TBG-XBP1s (1 × 10^12^ GC/mouse) through the jugular vein and the liver tissues of the mice were collected during feeding state at 16 days after the viral injection. To determine the nutritional status on the expression of the XBP1 gene, the liver tissues of three-month-old C57BL6 mice were collected during feeding state or after 24-h fast. In an experiment to assess XBP1u′s role in regulating glucose production, three-month-old C57BL6 mice were injected with AAV8-XBP1shRNA (shXBP1-3) (4 × 10ˆ11GC/mouse) and AAV8-TBG-GFP or AAV8-TBG-XBP1u (1.5 × 10^12^ GC/mouse) through the jugular vein (31), then the liver samples were harvested 12 days after the viral injection. A pyruvate tolerance test was conducted after 16 h of fast (1.5 g/kg).

### Glucose production assay and determination of liver triglyceride levels

Mouse primary hepatocytes were cultured in William’s medium E supplemented with ITS (BD Biosciences) and dexamethasone (32). After 16 h of planting, adenoviral shRNAs were added. Forty-eight hours later, primary hepatocytes were subjected to serum starvation for 3 h, followed by glucose production medium (20 mM lactate and 2 mM pyruvate) supplemented with 0.2 mM cAMP for another 3 h, and then both the medium and cells were collected. The medium was used to determine glucose concentrations with the EnzyChrom Glucose Assay Kit, and protein levels were determined in cellular lysates using the Bio-Rad Protein Assay Dye Kit. Cayman’s Triglyceride colorimetric assay kit was used to determine liver triglyceride levels following the steps recommended by the manufacturer. Briefly, 350 mg of liver tissues were minced and homogenized and centrifuged at 10,000*g* for 10 min. The supernatant was diluted (1:5), and 10 μl of standard and samples were added into 150 μm of enzyme mixture and incubated at room temperature for 15 min. The absorbance was determined at 530 nm.

### Immunoblots

Qiagen Tissue Homogenizer was used to homogenize the liver tissues (1 min, on ice). Cultured hepatocytes were harvested in Cell Lysis Buffer (Cell Signaling Technology). Cellular lysates were sonicated for 2 min and immunoblotted to examine the target proteins with antibodies against XBP1 (SC-7160, Santa Cruz, or #12782, Cell Signaling), phospho-AKTS473 (#9271), phospho-GSK3βS9 (#9336), phospho-MEK1/2S217/221 (#9154), phospho-ERKT202/204 (#4370), phospho-PERKT980 (#3179), phospho-eIF2αS51(#9721), phospho-p38T108/182 (#4631), eIF2α (#9722), IRΕ1 (#3294) (Cell signaling), and phospho-IRE1S724 (ab48187), ATF6 (ab37149), G6pc (ab83690), and PEPCK (ab70358) (abcam) at concentrations recommended by the manufacturers. Secondary antibodies were used at the concentrations around 1:5000 ∼ 10,000.

### Phosphorylation assay

For the phosphorylation of IRE1 by AKT1, 0.4 μg of human IRE1 (OriGene) was added to the reaction containing 25 mM Tris–HCl (pH7.5), 5 mM beta-glycerophosphate, 2 mM DTT, 0.1 mM Na_3_VO_4_, 10 mM MgCl_2_, and 0.1 μg of AKT1 (abcam) with or without 0.2 mM ATP. Samples were incubated at 37 °C for 1 h (33).

### Determination of the XBP1 splicing

TRIzol reagent was used to purify liver mRNA, and cDNA was synthesized using the iScript kit. The following primers were used to determine the splicing of XBP1: forward, 5′-TGACGAGGTTCCAGAGGT-3’; backward, 5′-GAGTCCATGGGAAGATGTTCTG-3’.

### Statistical analyses

Statistical significance was calculated with a Student’s *t* test and ANOVA test. Significance was accepted at the level of *p* < 0.05. At least three samples per group were chosen for statistically meaningful interpretation of results and differences in the studies using the Student’s *t* test and analysis of variation.

## Data availability

All data are contained in this manuscript and supporting information are available from the author: Ling He (heling@jhmi.edu) upon request.

## Conflicts of interest

The authors declare that they have no conflicts of interest with the contents of this article.
